# Exploring the ecological security evaluation of water resources in the Yangtze River Basin under the background of ecological sustainable development

**DOI:** 10.1038/s41598-024-65781-z

**Published:** 2024-07-05

**Authors:** Jie-Rong Zhou, Xiao-Qing Li, Xin Yu, Tian-Cheng Zhao, Wen-Xi Ruan

**Affiliations:** 1 Nanjing Xiaozhuang University, Nanjing, 211171 Jiangsu China; 2 School of Information Management, Nanjing University, Nanjing, 210023 Jiangsu China; 3 Wageningen University and Research, 6700 AA Wageningen, The Netherlands

**Keywords:** Lotka–Volterra symbiosis model, DPSIR model, Water resource ecological security, Yangtze River Basin, Evaluation system, Ecology, Environmental social sciences

## Abstract

The Yangtze River (hereafter referred to as the YZR), the largest river in China, is of paramount importance for ensuring water resource security. The Yangtze River Basin (hereafter referred to as the YRB) is one of the most densely populated areas in China, and complex human activities have a significant impact on the ecological security of water resources. Therefore, this paper employs theories related to ecological population evolution and the Driving Force-Pressure-State-Impact-Response (DPSIR) model to construct an indicator system for the ecological security of water resources in the YRB. The report evaluates the ecological security status of water resources in each province of the YRB from 2010 to 2019, clarifies the development trend of its water resource ecological security, and proposes corresponding strategies for regional ecological security and coordinated economic development. According to the results of the ecological population evolution competition model, the overall indicator of the ecological security of water resources in the YRB continues to improve, with the safety level increasing annually. Maintaining sound management of water resources in the YRB is crucial for sustainable socioeconomic development. To further promote the ecological security of water resources in the YRB and the coordinated development of the regional economy, this paper proposes policy suggestions such as promoting the continuous advancement of sustainable development projects, actively adjusting industrial structure, continuously enhancing public environmental awareness, and actively participating in international ecological construction and seeking cooperation among multiple departments.

## Introduction

Water is the primary resource for sustaining living organisms and also an important contributor to the ecological environment and the global economy. However, the current status of water resources is facing formidable challenges owing to rapid global population growth, sustained economic development, and extreme climatic conditions triggered by climate change. According to reports from the World Economic Forum and the United Nations, currently, over 2 billion people worldwide inhabit water-scarce regions, a figure projected to increase to as much as 3.5 billion by the year 2025. Approximately a quarter of the global population is confronting a “water stress” crisis, with water scarcity issues gradually becoming commonplace, defying prior expectations^1^. The report assessed the water risks in almost 200 countries and regions. Seventeen regions and countries around the world consume more than 80% of the available water supply, putting them at risk of experiencing severe water scarcity. The scarcity, uneven distribution, and deteriorating environmental quality of water resources have emerged as significant impediments to human sustainable development and societal progress, posing severe threats to water resource security across various regions. Consequently, there is an urgent imperative to engage in interdisciplinary research and foster collaborative innovation to devise scientifically sound water resource management strategies, thereby advancing the societal attainment of sustainable development goals.

Water resources are a strategic asset for ensuring economic and social development. Water is not only a fundamental element for human survival but also a crucial guarantee for economic and social development. If industry is the foundation of the national economy, then water is its “lifeblood”, essential for the development of all industries. As the largest river in China, the YZR originates from the Qinghai‒Tibet Plateau, traverses three major economic zones, and finally flows into the East China Sea. The YZR the world’s third-longest river and also has the widest basin area in China, accounting for approximately 36% of the country's total water resources. Thus, it is one of China’s most critical rivers. The YZR runs through eleven regions, including an autonomous region, eight provinces, and two municipalities directly under the central government, namely, Qinghai Province, the Tibet Autonomous Region, Yunnan Province, Sichuan Province, Hunan Province, Hubei Province, Jiangxi Province, Anhui Province, Jiangsu Province, Chongqing Municipality, and Shanghai Municipality. Due to the complex terrain and low population density in the Tibet Autonomous Region, human activities in the area have a relatively minor impact on water resource ecological security. Considering the integrity of administrative divisions, this paper selects ten provinces (municipalities), namely, Qinghai, Yunnan, Sichuan, Hunan, Hubei, Jiangxi, Anhui, Jiangsu, Chongqing, and Shanghai, as the research area, representing the YRB as the research object. The YRB currently has hundreds of millions of residents, meaning that the supply and demand of water resources in the basin are crucial for people’s livelihoods and industrial and agricultural production. As one of the most economically developed regions in China, the YRB has important economic centres and industrial bases. The rational utilization and management of water resources are crucial for the economic development of this region. Assessing the security of water resources in the YRB is the foundation for ensuring high-quality development in this area. To actively address the challenges posed by water security issues and achieve sustainable development, it is essential to prioritize and resolve water security challenges^2^.

By investigating research progress on water resource security both domestically and internationally, it has been found that the majority of studies primarily focus on the ecological system aspect, while a minority are based on the social attributes of water resources. Particularly within the realm of human–water relationships^3^, research examining the impact of socioeconomic factors on water resource ecological security from temporal and spatial perspectives is relatively limited. This study introduces the Lotka–Volterra biological concept to explore the competitive or symbiotic relationships between two populations concerning ecological resources within the same temporal and spatial context. Here, we assume that the changes in socioeconomic factors have an impact on the ecological security of water resources, and at the same time, the continuous improvement of water resource ecological security is also a sign of the advancement of socioeconomic development. The two mutually influence each other. Meanwhile, the water resource ecosystem possesses a certain degree of resilience, meaning that it can recover to a certain level through natural restoration or human intervention after being damaged to a certain extent. Building upon this foundation, the DPSIR model is employed to establish a symbiotic assessment index system for socioeconomic factors and water resources. The entropy weight method was utilized to calculate the weights of the indicators. Furthermore, the Lotka–Volterra coexistence model was employed to conduct an in-depth evaluation of the ecological security of water resources in the YRB from 2010 to 2019. The results indicate that during the period of 2010–2015, the ecological security status of water resources in the YRB was highly sensitive and even approached a dangerous state. However, with national governance and policy adjustments, the ecological security of water resources in the YRB has shown a trend of orderly recovery, currently stabilizing at a state of security or near-security. Nevertheless, challenges still exist in the management of water resource ecological security. It is vital not only to maintain and protect the YRB but also to further research and safeguard other water source areas. In summary, future efforts to govern and maintain the ecological security of water resources will be arduous, requiring the collaborative participation and governance of multiple stakeholders. Establishing a sound management system and calling for concerted efforts from the entire society to protect the YZR are crucial. Active participation in comprehensive ecological security protection projects in the YRB is essential. This lays the groundwork for constructing a healthier and more sustainable water resource ecological security management system.

## Research progress at domestic and abroad

### Interspecific competition model foundation—logistic model

The logistic curve, also known as the “S-shaped curve, ” is a graphical representation of the growth pattern of a population^4^. This logistic growth model was constructed by Verhulst^5^. The logistic model describes the development of many phenomena in nature, showing continuous growth within a certain period^6^. Generally, in the initial stages of species development, the population grows rapidly. After a certain period, the growth rate reaches its peak. Due to internal factors, the rate gradually slows until it no longer increases, reaching a stable state at the limit. This process of changing population size is referred to as a finite growth process, namely, the logistic growth process. According to the research results of scholars such as Haibo et al.^7^, Lingyun and Jun^8^, and Tao^9^, the basic interspecies competition model, the logistic model, is represented by the following equation:

1 $$\begin{array}{*{20}c} {\frac{{{\text{dN}}}}{{{\text{dt}}}} = \gamma N\left( {1 - \frac{{\text{N}}}{{\text{K}}}} \right)} \\ \end{array}$$

The constant ${\upgamma } > 0$ in the equation represents the self-intrinsic growth rate of the population, indicating the maximum growth rate of a single population without external environmental limitations. This variable reflects the difference between the average birth rate and the average death rate of individuals in a population who are not subjected to external inhibitory effects. This constant reveals the intrinsic growth characteristics of a species population. The parameter K reflects the abundance of available resources within an ecosystem. When the population size K of a species equals K, the population will no longer grow. Therefore, the K value represents the maximum number of individuals of a species that the ecosystem environment can accommodate, also known as the carrying capacity.

According to the logistic equation, we can observe that the relative growth rate of a population is proportional to the remaining resource capacity in the ecological system environment. When the remaining resources are abundant, the relative growth rate of the species population is high. This phenomenon, where the rate of population growth slows as population density gradually increases, is known as density-dependent regulation. As the ecological system capacity K approaches infinity, the growth rate of the population approaches exponential growth, and this change in the population growth curve is known as the logistic curve.

### Lotka–Volterra ecological model

In 1925, Lotka introduced a significant model in his research titled “Elements of Physical Biology”, the predator‒prey interaction model. This model quantitatively elucidates the interactions between organisms^10^. In 1926, Volterra, in his study “Variazionie fluttuazioni del numero d’individui in specie animali conviventi,” described the population dynamics of two interacting species in the biological realm^11^. These contributions laid the theoretical foundation for interspecific competition models and significantly influenced the development of modern ecological competition theories.

The interactions between species can be classified into three main types: competitive relationships, predator–prey relationships, and mutualistic cooperation relationships^12^. The Lotka–Volterra model was initially developed to describe predator‒prey relationships. However, with the increasingly widespread application of differential equation theory, this ecological model has evolved to encompass a broader range of applicability.

### DPSIR model

In 1993, the research group OECD innovatively proposed the DPSIR model, which is the “driving force-pressure-state-influence-response” model based on previous research models and has since been widely promoted in policy-making and research. Combining the characteristics of both the DSR (Driving Force-State-Response) and PSR frameworks, the DPSIR model effectively reflects causal relationships within systems, integrating elements such as resources, development, environment, and human health. As a result, it is considered a suitable method for evaluating watershed ecological security.

Consistent with the PSR framework, the DPSIR model organizes information and relevant indicators based on causal relationships with the aim of establishing a chain of causality: driving force (D)-pressure (P)-state (S)-impact (I)-response (R). In this context, “Driving Force (D)” primarily refers to potential factors reflecting changes in the health of the water cycle system, such as socioeconomic and population growth. “Pressure (P)” mainly refers to the impacts on the structure and functioning of the water cycle system, such as the utilization of water resources. “State (S)” represents changes in the water cycle system resulting from the combined effects of driving forces and pressures, serving as the starting point for impact and response analysis. “Impact (I)” reflects the effects of the hydrological cycle system on human health and social development. “Response (R)” refers to the feedback provided by the water cycle system to driving forces and pressures.

**Figure 1:**
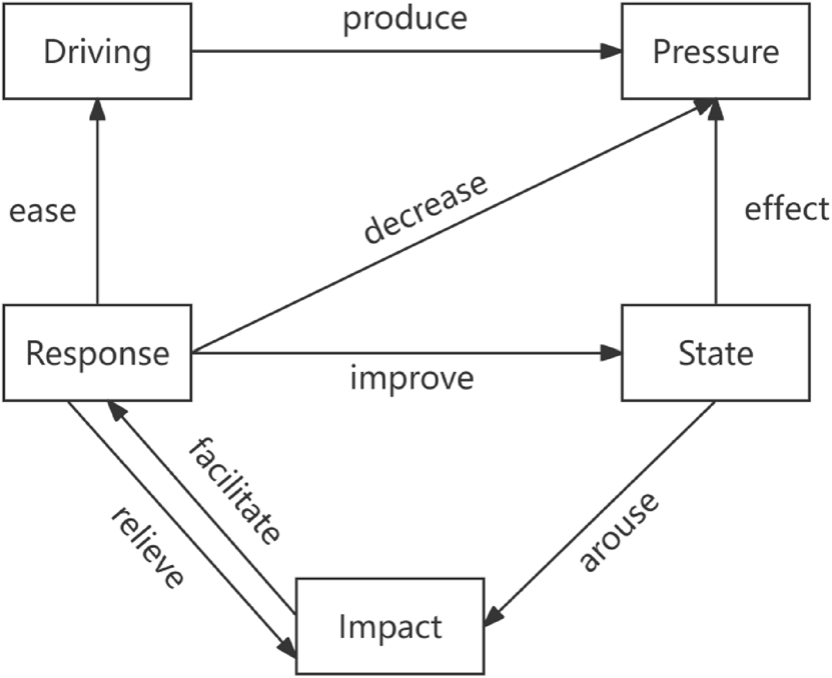
DPSIR model framework.

This model describes the causal chain between activities conducted by humans and the water environment, illustrating the mutually constraining and influencing processes between the two. It can encompass elements such as society, economy, and environment to indicate the threats posed by social, economic, and human activities to watershed ecological security. It can also utilize response indicators to demonstrate the feedback of the environment to society resulting from human activities and their impacts, as shown in Fig. 1^13^.

### Overview of water resource ecological security

Water resources are a vital strategic asset for sustainable development and a key factor influencing human survival and socioeconomic development. The security of water resources is intricately linked to national economies and social stability^14–18^. As the population and economy grow rapidly, as well as due to the influence of climate change, water scarcity and deterioration of the water environment have become increasingly prevalent, posing a critical constraint to human survival and development^19^. Currently, research on water resource ecological security issues primarily revolves around the following three aspects.

The first aspect involves the evaluation of the water resources carrying capacity (hereafter referred to as the WRCC) and vulnerability.

Regarding the WRCC, some studies consider that the WRCC implies the need for water resources to sustain a healthy societal system^20^. Other researchers argue that the WRCC is the maximum threshold for sustaining human activities^21^.

In terms of calculation methods, various quantification methods for the WRCC have gradually emerged. For example, Qu and Fan^22^ considered the available water volume in water demand, national economic sectors and the ecological environment. They employed the traditional trend approach to obtain the population and development scales of industry and agriculture. Zhou Fulei adopted the entropy weight method, an objective weight determination method, to determine the weights of each evaluation indicator, utilized the analytic hierarchy process (AHP) to adjust the weights, constructed composite weights, and then used the TOPSIS model to evaluate the water resources carrying capacity of Qingdao city from 2015 to 2021^23^. Ma et al.^24^and Xiong et al.^25^ analysed and evaluated the WRCC using the entropy weight method and provided suggestions for regional sustainable development. Wang et al.^26^, under the traditional TOPSIS model, used an improved structural entropy weighting method to determine the weights of evaluation indicators. They then constructed a grey-weighted TOPSIS model using a grey correlation matrix to specifically evaluate the current state of the agricultural WRCC in Anhui Province. Zhang X and Duan X combined the weights obtained from the entropy and CRITIC methods using the geometric mean method. They applied these combined weights to a model integrating grey relational analysis (GRA), the technique for order preference by similarity to an ideal solution (TOPSIS), and the coupling coordination degree model (CCDM) to calculate the evaluation value of the water resource carrying capacity^27^. Zhang and Tan^28^ and Fu et al.^29^ separately used optimization models and projection tracking models to evaluate the WRCC in their study areas and conducted comprehensive assessments of the regional WRCC. Gong and Jin^30^, Meng et al.^31^, Wang et al.^32^, and Gao et al.^33^ applied fuzzy comprehensive evaluation methods to assess the influencing factors of the WRCC by establishing a fuzzy comprehensive evaluation matrix. On this basis, they analysed the factors affecting the WRCC and evaluated and predicted the future carrying capacity of water resources in the study area. Additionally, other methods have been employed, such as multidimensional regulation^34^, neural network genetic algorithms^35,36^, multi-index evaluation models^37^, and nonparametric analysis models^38^.

Ait-Aoudia and Berezowska-Azzag^39^ conducted an assessment of the WRCC to analyse the balance between domestic demand and water supply. To assess the WRCC of specific regions, the assessment factors were determined by evaluating the relevant factors of water usage and availability. The conceptual framework for assessing the capacity of water resources was developed based on the supply–demand relationship. Yan et al.^40^ focused on the previous decade’s regional water resource data of Anhui Province in China. They constructed a framework for the Driving Force-Pressure-State-Impact-Response Management (DPSIRM) model and conducted a comprehensive evaluation of the WRCC using the entropy weight method and variable weight theory. Based on the derived comprehensive evaluation values and incorporating the modified Gray–Markov combined forecasting, they made predictions about the local WRCC for the coming years. In 2020, Zhengqian^41^ discussed the concept and research methods of regional WRCC. The research methodology has evolved from a singular and static approach to a dynamic, multilevel, and comprehensive study with various indicators. Jiajun et al.^42^, starting from a systemic perspective, studied the coordinated development relationships among China’s economy, social development, ecological environment, and water resources. They applied the WRCC Comprehensive Evaluation Model, calculating the comprehensive evaluation index for specific years based on relevant data. This allowed them to describe the WRCC status of provinces and regions in China, providing a comprehensive analysis and evaluation of China’s WRCC. Ren et al.^43^ introduced the concept of biological metabolism to the regional WRCC and proposed the theory of regional water resource metabolism. Additionally, they established an evaluation indicator system for the WRCC considering regional water resource characteristics, socioeconomic systems, and sustainable development principles.

Raskin et al.^44^ assessed the extent of water resource security by using the proportion of water extraction relative to the total water resources, defined as the water resource vulnerability index. Rui^45^ constructed a water resource vulnerability model based on the theory of mutation series. They utilized the principles of mutation series to redefine grading standards and assessed the vulnerability status of water resources in Shanxi Province from 2004 to 2016. The aim was to offer technical assistance for the scientific management of water resources.

The second aspect involves the measurement of the sustainable utilization and efficiency of regional water resources.

Over the last few years, numerous domestic researchers have actively conducted research on the sustainable utilization of water resources, focusing primarily on two aspects:

First, research on evaluation indicator systems for the sustainable utilization of water resources should be conducted. Li Zhijun, Xiang Yang, and others addressed the lack of connection between water resource ecology and socioeconomic development in traditional water resource ecological footprint methods. They introduced the water resource ecological benefit ratio and analysed the water resource security and sustainable development status through an improved water resource energy value ecological footprint method^46^. Zhang et al.^47^ established a fuzzy comprehensive evaluation model based on entropy weight, providing recommendations for the sustainable utilization of water resources in Guangxi Province. Liu Miliang, aiming for sustainable development, quantitatively analysed the current situation and influencing factors. Based on the DPSIR model, they established an evaluation system for the sustainable utilization of water resources^48^.

Second, in terms of evaluation methods and research on the sustainable utilization of water resources, Yunling et al.^49^ constructed an evaluation indicator system for the WRCC to assess the comprehensive water resource carrying status in Hebei Province. Xuexiu et al.^50^, based on both domestic and international research on water resource pressure theory, analysed the connotation of water resource pressure, introduced commonly used methods for water resource pressure evaluation, and provided a comprehensive overview and comparative analysis of water resource pressure evaluation methods from aspects such as calculation principles, processes, and applications. Guohua et al.^51^ established an entropy-based fuzzy comprehensive evaluation model of water resource allocation harmony and evaluated the water resource allocation status of various districts and counties in Xi’an city. Shiklomanov^52^ used indicators such as available water resources, industrial and agricultural water usage, and household water consumption to assess water resource security.

The SBM-DEA model was used by Deng et al.^53^ to appraise the efficiency of water resource utilization across nearly all provinces in China. They proposed factors influencing water resource utilization efficiency, including the added value of the agricultural sector, per capita water usage, the output-to-pollution ratio of polluting units, and import–export dependency. Yaguai and Lingyan^54^ employed a two-stage model combining superefficiency DEA and Tobit to assess water resource efficiency in China from 2004 to 2014. They analysed regional differences and influencing factors. Mei et al.^55^ separately used stochastic frontier analysis and data envelopment analysis to measure the absolute and relative efficiencies of water resource utilization in 14 cities in Liaoning Province. They employed a kernel density estimation model to analyse the dynamic evolution patterns of water resource utilization efficiency. Xiong et al.^56^ adopted an iterative correction approach to modify and apply water resource utilization efficiency evaluation models based on single assessment methods such as entropy, mean square deviation, and deviation methods.

The third aspect involves investigating the relationship between water resource security and other societal systems.

Shanshan et al.^57^ laid the foundation for the rational construction of an urbanization and water resource indicator system. Through the establishment of a dynamic coupled model, they conducted an analytical study on the harmonized development trends between the urbanization system and the water resource system in Beijing. Wei^58^ utilized a coordination degree model to explore the coupling relationship between the quality of new urbanization and water resource security in Guangdong Province. Caizhi and Xiaodong^59^ combining coupled scheduling models with exploratory spatial data analysis and conducted an analysis of the security conditions and spatial correlations among water resources, energy, and food in China. Additionally, Xia et al.^60^ employed the Mann–Kendal test method to study the degrees of matching between water resources and socioeconomic development in six major geographical regions of China.

A review of the relevant literature reveals that scholars have explored the issues of water resource ecological security and regional socioeconomic development from various perspectives and fields, which is one of the urgent problems to be addressed in the current process of social development. These research findings not only have learning and reference significance but also provide insights for the writing of this paper.

Summarizing the achievements of previous research, the essence of water resource security evaluation mainly includes three aspects: ensuring water quantity, sustainability, and water quality. Evaluation methods include principal component analysis, fuzzy comprehensive evaluation methods, analytic hierarchy processes, and system dynamics modelling methods, among others, among which the analytic hierarchy process has certain advantages in addressing multilevel problems and is widely used in constructing multilevel analysis models. Therefore, this paper introduces the Lotka–Volterra biological concept and continues to explore this topic further. It can effectively combine the relationships between indicators and weights and study the competition or symbiotic relationship between two populations competing for ecological resources in the same time and space context^61^. Drawing from the DPSIR model, this study devises a comprehensive evaluation framework to assess the interdependence of socioeconomic factors and water resources. Through the application of the entropy weight method, this study determines the relative importance of various indices within this framework. Employing the Lotka–Volterra symbiotic model, this research scrutinizes and quantifies the ecological security status of water resources in the YRB from 2010 to 2019. The overarching objective is to furnish technical insights that can catalyse efforts to enhance the ecological security of regional water resources.

## Methodology

### Lotka–Volterra symbiosis model

In the 1940s, A. J. Lotka and V. Volterra jointly introduced the Lotka–Volterra model^62^, which serves as a method for studying the relationships between biological populations. Its basic form is as follows:

2 $$\begin{array}{*{20}c} {\frac{{{\text{dN}}_{1} \left( {\text{t}} \right)}}{{{\text{dt}}}} = {\text{r}}_{1} {\text{N}}_{1} \left( {\text{t}} \right)\frac{{{\text{K}}_{1} - {\text{N}}_{1} \left( {\text{t}} \right) - \alpha {\text{N}}_{2} \left( {\text{t}} \right)}}{{{\text{K}}_{1} }}} \\ \end{array}$$

3 $$\begin{array}{*{20}c} {\frac{{{\text{dN}}_{2} \left( {\text{t}} \right)}}{{{\text{dt}}}} = {\text{r}}_{2} {\text{N}}_{2} \left( {\text{t}} \right)\frac{{{\text{K}}_{2} - {\text{N}}_{2} \left( {\text{t}} \right) - \alpha {\text{N}}_{2} \left( {\text{t}} \right)}}{{{\text{K}}_{2} }}} \\ \end{array}$$

In the given equation, ${\text{N}}_{1} \left( {\text{t}} \right), {\text{N}}_{2} \left( {\text{t}} \right)$ denote the populations of species ${\text{S}}_{1}$ and ${\text{S}}_{2}$, respectively. ${\text{K}}_{1}$ and ${\text{K}}_{2}$ represent the carrying capacities of populations ${\text{S}}_{1}$ and ${\text{S}}_{2}$ in their respective environments. ${\text{r}}_{1}$ and ${\text{r}}_{2}$ represent the growth rates of populations ${\text{S}}_{1}$ and ${\text{S}}_{2}$, respectively. $\alpha$ denotes the competitive intensity coefficient of species ${\text{S}}_{2}$ on species ${\text{S}}_{1}$, while $\beta$ represents the competitive intensity coefficient of species ${\text{S}}_{1}$ on species ${\text{S}}_{2}$.

By replacing the socioeconomic relationships within the entire YRB with the provinces within the basin, the Lotka–Volterra model is introduced into the regional water resource ecological security assessment. This allows for the construction of a symbiotic model between socioeconomic factors and water resources within the YRB. The specific formula is as follows:

4 $$\begin{array}{*{20}c} {\frac{{{\text{dF}}\left( {\text{k}} \right)}}{{{\text{dk}}}} = {\text{r}}_{{\text{F}}} F\left( {\text{k}} \right)\frac{{{\text{C}} - {\text{F}}\left( {\text{k}} \right) - \alpha {\text{E}}\left( {\text{k}} \right)}}{{\text{C}}}} \\ \end{array}$$

5 $$\begin{array}{*{20}c} {\frac{{{\text{dE}}\left( {\text{k}} \right)}}{{{\text{dk}}}} = {\text{r}}_{{\text{E}}} E\left( {\text{k}} \right)\frac{{{\text{C}} - {\text{E}}\left( {\text{k}} \right) - \beta {\text{F}}\left( {\text{k}} \right)}}{{\text{C}}}} \\ \end{array}$$

In the equation, ${\text{F}}\left( {\text{k}} \right)$ denotes the comprehensive socioeconomic development status, ${\text{E}}\left( {\text{k}} \right)$ signifies the comprehensive development status of water resources, ${\text{C}}$ represents the ecological environment, ${\text{r}}_{{\text{F}}}$ signifies the socioeconomic growth rate, ${\text{r}}_{{\text{E}}}$ represents the growth rate of water resources, $\alpha$ denotes the coefficient of water resources’ impact on the socioeconomy, and $\beta$ denotes the coefficient of the impact of the socioeconomy on water resources. Therefore, solving for the coefficients $\alpha$ and $\beta$ in the model is essential for examining the interaction between the socioeconomy and water resources. The specific steps for solving the equation are as follows.

Discretizing Eqs. (4), (5) yields:

6 $$\begin{array}{*{20}c} {F\left( {{\text{k}} + 1} \right) - F\left( {\text{k}} \right) = \frac{{{\text{F}}\left( {\text{k}} \right) - {\text{F}}\left( {{\text{k}} - 1} \right)}}{{{\text{F}}\left( {{\text{k}} - 1} \right)}} \cdot F\left( {\text{k}} \right)\frac{{{\text{C}}\left( {\text{k}} \right) - {\text{F}}\left( {\text{k}} \right) - \alpha \left( {\text{k}} \right){\text{E}}\left( {\text{k}} \right)}}{{{\text{C}}\left( {\text{k}} \right)}}} \\ \end{array}$$

7 $$\begin{array}{*{20}c} {E\left( {{\text{k}} + 1} \right) - E\left( {\text{k}} \right) = \frac{{{\text{E}}\left( {\text{k}} \right) - {\text{E}}\left( {{\text{k}} - 1} \right)}}{{{\text{E}}\left( {{\text{k}} - 1} \right)}} \cdot E\left( {\text{k}} \right)\frac{{{\text{C}}\left( {\text{k}} \right) - {\text{E}}\left( {\text{k}} \right) - \beta \left( {\text{k}} \right){\text{F}}\left( {\text{k}} \right)}}{{{\text{C}}\left( {\text{k}} \right)}}} \\ \end{array}$$

The solution is:

8 $$\begin{array}{*{20}c} {\alpha \left( {\text{K}} \right) = \frac{{\varphi_{{\text{F}}} \left( {\text{k}} \right){\text{C}}\left( {\text{k}} \right) - {\text{F}}\left( {\text{k}} \right)}}{{{\text{E}}\left( {\text{k}} \right)}}} \\ \end{array}$$

9 $$\begin{array}{*{20}c} {\beta \left( {\text{K}} \right) = \frac{{\varphi_{{\text{E}}} \left( {\text{k}} \right){\text{C}}\left( {\text{k}} \right) - {\text{E}}\left( {\text{k}} \right)}}{{{\text{F}}\left( {\text{k}} \right)}}} \\ \end{array}$$

In which:

10 $$\begin{array}{*{20}c} {\varphi_{{\text{F}}} \left( {\text{k}} \right) = 1 - \frac{{{\text{F}}\left( {{\text{k}} + 1} \right) - {\text{F}}\left( {\text{k}} \right)}}{{{\text{F}}\left( {\text{k}} \right)}} \cdot \frac{{{\text{F}}\left( {{\text{k}} - 1} \right)}}{{{\text{F}}\left( {\text{k}} \right) - {\text{F}}\left( {{\text{k}} - 1} \right)}} = 1 - \frac{{{\text{r}}_{{\text{F}}} \left( {{\text{k}} + 1} \right)}}{{{\text{r}}_{{\text{F}}} \left( {\text{k}} \right)}}} \\ \end{array}$$

11 $$\begin{array}{*{20}c} {\varphi_{{\text{E}}} \left( {\text{k}} \right) = 1 - \frac{{{\text{E}}\left( {{\text{k}} + 1} \right) - {\text{E}}\left( {\text{k}} \right)}}{{{\text{E}}\left( {\text{k}} \right)}} \cdot \frac{{{\text{E}}\left( {{\text{k}} - 1} \right)}}{{{\text{E}}\left( {\text{k}} \right) - {\text{E}}\left( {{\text{k}} - 1} \right)}} = 1 - \frac{{{\text{r}}_{{\text{E}}} \left( {{\text{k}} + 1} \right)}}{{{\text{r}}_{{\text{E}}} \left( {\text{k}} \right)}}} \\ \end{array}$$

**Figure 2:**
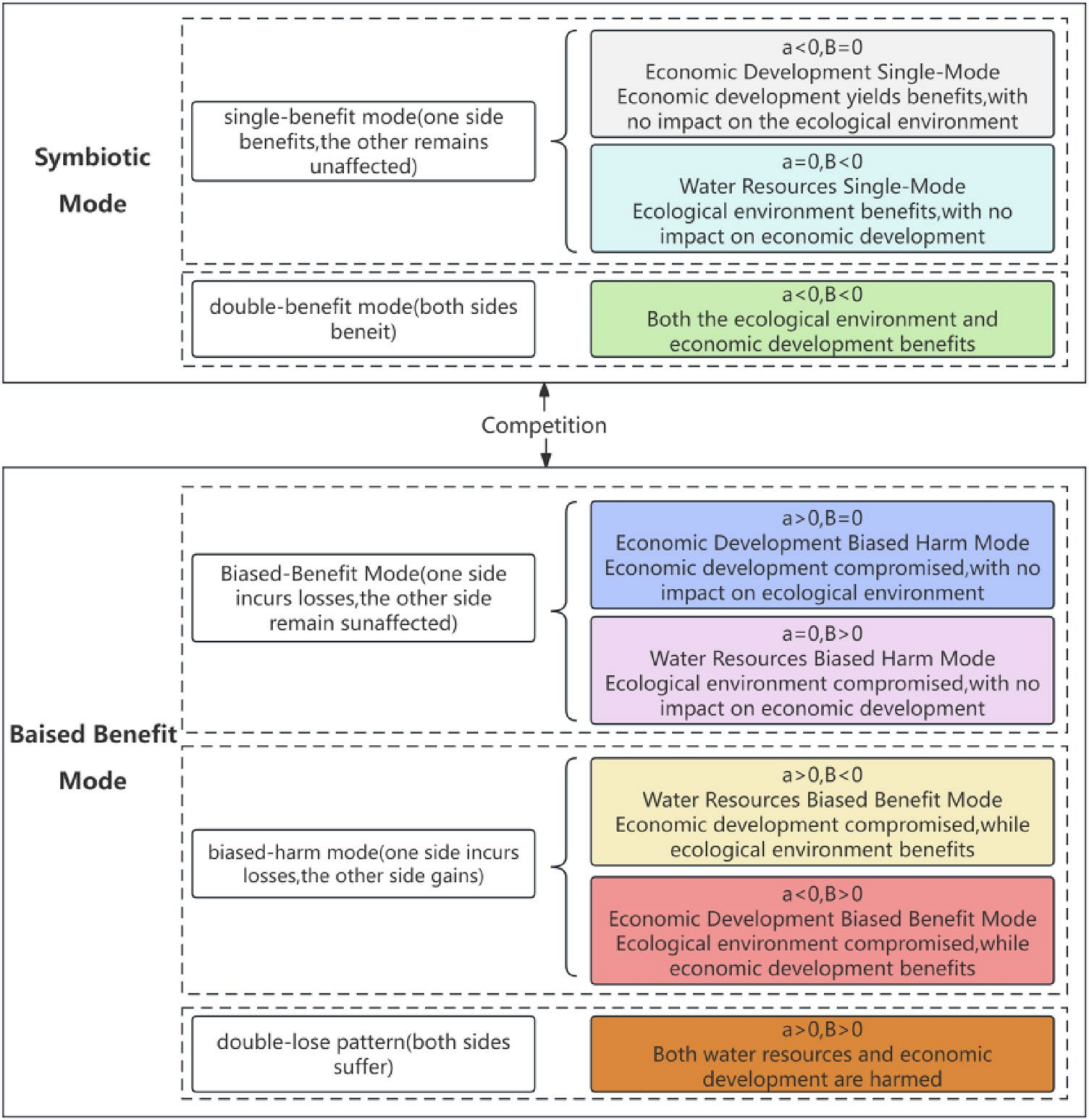
Symbiotic model between the socioeconomic and water resources in the YRB.

Different values of $\alpha$ and $\beta$ correspond to different symbiotic relationships between the socioeconomy and water resources, as illustrated in Fig. 2.

### Construction of the DPSIR model and indicator system

To construct a water resource ecological security index system for the 10 provinces in the YRB, this paper is based on the research of relevant scholars and introduces the DPSIR model to evaluate water resource ecological security. This model was proposed to describe the concept of environmental systems and the structure of complex cause-and-effect relationships by the European Environment Agency (EEA) in 1999. It is mainly applied in assessments of ecological security, regional sustainable development, and water resource ecological security.

**Figure 3:**
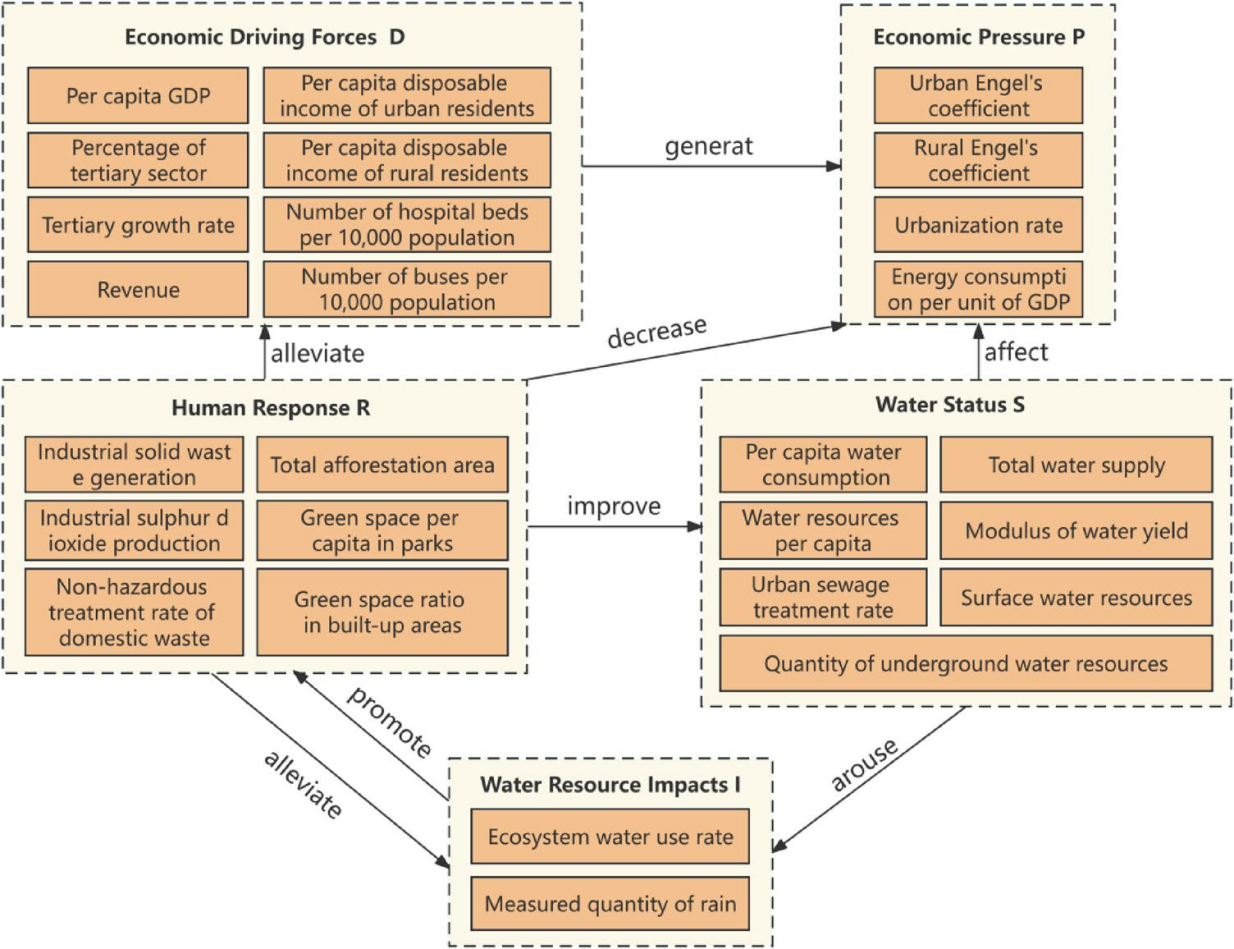
DPSIR model.

The establishment of the DPSIR model in this paper is illustrated in Fig. 3.

Generally, the driver (D) in the socioeconomic system tends to improve the environmental and resource states (S), while the economic pressure (P) tends to disrupt the resource and environmental states (S). The states of resources and the environment contribute essential production materials to the socioeconomic system. Simultaneously, drivers (D) and pressures (P) reflect two different aspects of socioeconomic development. Therefore, these factors can indicate the level of socioeconomic development. Based on these definitions, the following indicators are selected to assess the DPSIR model for water resource ecological security. The weights of various indicators calculated through the entropy weight method are presented in Table 1. A more significant role played by the corresponding indicator in the comprehensive assessment of regional ecological security will have a greater weight.

**Table 1:** Index construction and index weights of the water resource ecological security DPSIR model.

Target level	Criterion level		Indicator level		Nature of indicators	Indicator weight
Socioeconomic F	Economic Driving Force D		Per Capita GDP		+	0.0480
			Proportion of the Tertiary Industry		+	0.0474
			Growth Rate of the Tertiary Industry		+	0.0153
			Fiscal Revenue		+	0.0491
			Per Capita Disposable Income of Urban Residents		+	0.0461
			Per Capita Disposable Income of Rural Residents		+	0.0415
			Number of Hospital Beds per Ten Thousand People		+	0.0364
			Number of Cars per Ten Thousand People		+	0.0521
	Economic Pressure P		Urban Engel Coefficient		−	0.0321
			Rural Engel Coefficient		−	0.0237
			Urbanization Rate		−	0.0209
			Energy Consumption per Unit of Gross Domestic Product (GDP)		−	0.0106
Ecological Environment C	Water Resource E		Resource status S	Per Capita Water Consumption	−	0.0191
				Per Capita Water Resources	+	0.0900
				Urban Sewage Treatment Rate	+	0.0055
				Surface Water Resources	+	0.0516
				Groundwater Resources	+	0.0480
				Total Water Supply	+	0.0492
				Water Production Module	+	0.0973
			Resource Impact I	Ecological Environment Water Usage Rate	+	0.0617
				Precipitation	+	0.0227
	Human Response R		Industrial Solid Waste Generation		−	0.0205
			Industrial Sulfur Dioxide Emission		−	0.0028
			Harmless Treatment Rate of Domestic Waste		+	0.0113
			Total Afforested Area		+	0.0544
			Per Capita Park and Green Space Area		+	0.0289
			Green Space Ratio in Built-up Areas		+	0.0138

Note: When studying the relationship between socioeconomy and water resources, it is crucial to recognize that water resources are an important component of the ecological environment. Therefore, we consider water resources as part of the ecological environment subsystem when constructing the evaluation index system. This means that water resources are not only managed and utilized economically but also influenced by their impact on ecosystem health and the ecosystem environment. Through this system of comprehensive evaluation indices, socioeconomic activities, water resources, and the natural environment can be considered more fully.

On this basis, the socioeconomic stress index ${\text{S}}_{{\text{F}}} \left( {\text{k}} \right)$ and water resource stress index ${\text{S}}_{{\text{E}}} \left( {\text{k}} \right)$ are defined as follows:

12 $$\begin{array}{*{20}c} {{\text{S}}_{{\text{F}}} \left( {\text{k}} \right) = - \alpha \left( {\text{k}} \right) = - \frac{{({{\varphi }}_{{\text{F}}} \left( {\text{k}} \right){\text{C}}\left( {\text{k}} \right) - {\text{F}}\left( {\text{k}} \right))}}{{{\text{E}}\left( {\text{k}} \right)}}} \\ \end{array}$$

13 $$\begin{array}{*{20}c} {{\text{S}}_{{\text{E}}} \left( {\text{k}} \right) = - \beta \left( {\text{k}} \right) = - \frac{{({{\varphi }}_{{\text{E}}} \left( {\text{k}} \right){\text{C}}\left( {\text{k}} \right) - {\text{E}}\left( {\text{k}} \right))}}{{{\text{F}}\left( {\text{k}} \right)}}} \\ \end{array}$$

The comprehensive index between socioeconomic and water resources, also called the symbiosis index ${\text{S}}\left( {\text{k}} \right)$, is calculated as follows:

14 $$\begin{array}{*{20}c} {S\left( {\text{k}} \right) = \frac{{{\text{S}}_{{\text{F}}} \left( {\text{k}} \right) + {\text{S}}_{{\text{E}}} \left( {\text{k}} \right)}}{{\sqrt {{\text{S}}_{{\text{F}}}^{2} \left( {\text{k}} \right) + {\text{S}}_{{\text{E}}}^{2} \left( {\text{k}} \right)} }}} \\ \end{array}$$

According to Eq. (14), ${\text{S}}\left( {\text{k}} \right) \in \left[ { - \sqrt 2 ,\sqrt 2 } \right]$, a larger value of A indicates that the symbiotic state between the socioeconomy and water resources is better; conversely, a smaller value of A indicates that the symbiotic state between the two is worse.

The water resources force index can illustrate the direction of the socioeconomic impact on water resources, and the symbiotic index can illustrate the magnitude of the socioeconomic impact on water resources. Therefore, these two indices serve as the basis for evaluating the water resource security status. Formula (14) implies that the symbiotic index ${\text{S}}\left( {\text{k}} \right)$ falls within the range of $\left[ { - \sqrt 2 ,\sqrt 2 } \right]$. A larger numerical value indicates a better symbiotic relationship between the two subsystems, while a smaller value suggests a poorer symbiotic relationship. However, the relationship between the symbiotic index and regional ecological security is not straightforward. Regional ecological security must be judged according to specific criteria grounded in both the measure of symbiosis ${\text{S}}\left( {\text{k}} \right)$ and the ecological force index ${\text{S}}_{{\text{E}}} \left( {\text{k}} \right)$. This approach comprehensively characterizes the ecological security of the YRB urban agglomeration. In our study, a two-dimensional symbiotic model of socioeconomic–natural ecology is employed to depict the evolution of ecological security under dual-characteristic indices.

Within this model, ecological security is divided into six regions that progress in a sequential manner, conforming to the progressive law of ecological security evolution. In the safe zone, the socioeconomic and natural ecological systems mutually benefit, and both experience robust development. In the subsafe zone, although the natural ecological system is still in a growing state, this occurs at the expense of socioeconomic development, leading to an unstable ecological security status. If the socioeconomic system continues to suffer damage, it falls into the sensitive zone, where the harm to the socioeconomic system outweighs the benefits to the natural ecological system. If this condition persists, both systems enter a state of competition, resulting in harm to both, and they are situated in the danger zone. In unfavourable zones, the socioeconomic system gains weak benefits, while the natural economy suffers damage. If humanity recognizes this situation and takes measures to improve the environment, it may transition from the unfavourable zone to the cautious zone, leading to an improvement in ecological security and potential entry into the safe zone. For ease of analysis and based on the relevant literature^63^, following expert discussions, this study classifies ecological security into six categories corresponding to six ecological security early warning levels, as shown in Table 2.

**Table 2:** Water resource ecological security early warning level table.

Serial number	Water resource stress index	Symbiosis index	Symbiotic relationship	Water resource security status	Ecological security level
1	SE(k) > 0	1 < S(k) < √2	Mutually Beneficial Symbiosis between Economic and Water Resources	Safe	I
2	SE(k) > 0	0 < S(k) < 1	Economic Losses, Water Resources Gains	Subsecure	II
3	SE(k) < 0	0 < S(k) < 1	Economic Gains, Water Resources Losses	Vigilant	III
4	SE(k) > 0	− 1 < S(k) < 0	Economic Losses, Water Resources Gains	Sensitive	IV
5	SE(k) < 0	− 1 < S(k) < 0	Economic Gains, Water Resources Losses	Deteriorating	V
6	SE(k) < 0	− √2 < S(k) < − 1	Economic Losses, Water Resources Gains	Dangerous	VI

### Discrimination of water resource ecological security levels

The YZR originates from the Qinghai‒Tibet Plateau, considered the “Roof of the World,” traversing three major economic regions before ultimately flowing into the East China Sea. For our study area, we selected the eight provinces and two municipalities through which the YZR flows. These regions are Shanghai, Jiangsu, Anhui, Jiangxi, Hubei, Hunan, Chongqing, Sichuan, Yunnan, and Qinghai. In the subsequent text, they will be referred to collectively as the YRB. The data for this study primarily originate from statistical yearbooks, water resource bulletins, and development reports spanning the years 2010 to 2019.

According to the criteria for water resource security status presented in Table 2, the corresponding information is summarized in Table 3 for the years 2011 to 2018, indicating the water resource security status in the YRB during this period. It is observed that from 2011 to 2018, the water resources security status in the YRB initially experienced a decline but later recovered to a secure level. In recent years, the country has not only emphasized economic development but also placed significant importance on environmental protection. Rapid industrial development in earlier years led to an exacerbation of water pollution issues. However, the government promptly recognized this problem and implemented a series of measures to address water pollution. Stringent controls were also imposed on industrial water usage. Consequently, the water resource status quickly returned to a level considered safe.

**Table 3:** Table of water resource security status in the YRB from 2011 to 2018.

Year	Symbiosis Index	Water resource security status	Level
2011	0 < S(k) < 1	Vigilant	III
2012	− √2 < S(k) < − 1	Dangerous	VI
2013	0 < S(k) < 1	Vigilant	III
2014	− 1 < S(k) < 0	Sensitive	IV
2015	− 1 < S(k) < 0	Deteriorating	V
2016	− 1 < S(k) < 0	Sensitive	IV
2017	1 < S(k) < √2	Safe	I
2018	1 < S(k) < √2	Safe	I

The water resource security evaluation values obtained using the entropy method range from 0 to 1. Ideally, a value closer to 1 indicates a better water resource security situation, while a value closer to 0 suggests a poorer water resource security situation.

After standardizing the processed data, we can plug them into Eq. (15) to sequentially obtain the basic indices for socioeconomic, ecological environment, and water resource security in the YRB. The specific process involves substituting the basic indices for socioeconomic, ecological environment, and water resource ecological security into Eqs. (12)–(14). This approach yields comprehensive indices, including the socioeconomic stress index, water resource stress index, and symbiotic degree index. These indices serve as the basis for evaluating the water resource security status in the assessment region, with the water resource stress index and symbiotic degree index being the key indicators.

15 $$\begin{array}{*{20}c} {{\text{f}}_{{\text{i}}} = \mathop \sum \limits_{{{\text{i}} = 1}}^{{\text{n}}} {\text{x}}_{{\text{i}}}^{\prime } {\text{w}}_{{\text{i}}} } \\ \end{array}$$

In the equation, f_i_ represents the comprehensive level of water resource ecological security, ${\text{x}}_{{\text{i}}}^{\prime }$ signifies the standardized values obtained from the original data, and ${\text{w}}_{{\text{i}}}$ denotes the weights assigned to each indicator. When the value of f_i_ falls between 0 and 1, the closer the value is to 1, the better the ecological security of water resources. In contrast, it shows a poorer ecological security status. Similarly, according to this equation, the classification of water resource ecological security can be divided into six categories: 0–0.16 denotes a dangerous state, 0.16–0.32 indicates a deteriorating state, 0.32–0.48 signifies a sensitive state, 0.48–0.64 represents a vigilant state, 0.64–0.8 implies a subsecure state, and 0.8–1.0 corresponds to a safe state. Different levels of water resource ecological security entail varying relationships with the national economy and society. For specific characteristics corresponding to each security level, please refer to Table 4.

**Table 4:** Classification of water resource security status in the YRB.

Assessment value	Level	Status	Feature
0 ≤ fi < 0.16	VI	Dangerous	Water resources are severely scarce, hindering the sustainable development of the national economy and society
0.16 ≤ fi < 0.32	V	Deteriorating	Water resources are scarce and cannot meet the needs of sustainable development for the national economy and society
0.32 ≤ fi < 0.48	IV	Sensitive	Water resources are slightly scarce, barely meeting the needs of sustainable development for the national economy and society
0.48 ≤ fi < 0.64	III	Vigilant	Water resources can essentially meet the needs of sustainable development for the national economy and society
0.64 ≤ fi < 0.8	II	Subsecure	Water resources can meet the needs of sustainable development for the national economy and society, reaching a satisfactory state
0.8 ≤ fi < 1.0	I	Safe	Water resources completely meet the needs of sustainable development for the national economy and society, achieving an ideal state

### Informed consent statement

Informed consent was obtained from all subjects involved in the study.

## Results

### Evaluation of water resource ecological security levels in the Yangtze River Basin

Overall, the evaluation values of water resource security in the YRB from 2010 to 2019 showed a fluctuating upwards trend (refer to Table 5). From 2010 to 2013, the evaluation values fluctuated between 0.2 and 0.4, reaching the lowest level at Grade V. In 2011, the evaluation value was only 0.2201, indicating that during this period, the water resources in the YRB were in an unsafe state, resulting in water scarcity. These results indicate that economic and social development are not being met on a sustainable basis at the watershed scale. In 2014, the water resource security evaluation value for the YRB reached 0.4243, classified as Grade III. Subsequently, there was a significant upwards trend, with the evaluation value reaching 0.6746 in 2017, which was classified as Grade II, indicating a relatively secure state. These results suggest that the water resources of the YRB appeared to be more secure than they were before, and the YRB could essentially fulfil the requirements for sustainable economic and social development at the national level. This upwards trend continued, reaching 0.7215 in 2019. From 2010 to 2019, the water resource security status in the YRB improved from Grade V to Grade II, demonstrating significant improvement. However, it has not yet reached Grade I, indicating that there is still room for improvement in the future.

**Table 5:** Evaluation results of water resource ecological security in the YRB from 2010 to 2019.

Year	Criterion-level assessment value					Appraisal value	Level
	Motivating force	Pressure	Status	Impact	Response		
2010	0.0543	0.0403	0.1633	0.0261	0.0527	0.3368	IV
2011	0.0816	0.0392	0.0200	0.0257	0.0536	0.2201	V
2012	0.0788	0.0447	0.1867	0.0158	0.0605	0.3865	IV
2013	0.0926	0.0568	0.0656	0.0231	0.0698	0.3079	V
2014	0.1100	0.0925	0.1278	0.0208	0.0732	0.4243	IV
2015	0.1511	0.0956	0.1111	0.0292	0.1124	0.4994	III
2016	0.1745	0.0992	0.1755	0.0436	0.0978	0.5907	III
2017	0.2036	0.1057	0.1712	0.0346	0.1594	0.6746	II
2018	0.2195	0.1170	0.1625	0.0363	0.1585	0.6938	II
2019	0.2370	0.1149	0.1682	0.0349	0.1665	0.7215	II

The DPSIR model was used to analyse the reasons for the improvement in the ecological security of water resources in the YRB based on five criteria. Table 5 shows that the evaluation values for driving forces significantly increased from 2010 to 2019, while the values for pressure and response slightly increased, and those for state and impact fluctuated, resulting in a slight overall improvement. Specifically, the evaluation values for driving forces fluctuated from 0.0543 to 0.2370, indicating the significant contributions of indicators such as per capita GDP, the proportion of primary industry, population density, and the urbanization rate to the enhancement of water resource security. The assurance provided by economic and social development for water resource security is evident. The evaluation value for pressure fluctuated from 0.0403 to 0.1149, suggesting a reduction in pressure on water resources from economic development, agricultural and industrial production, and residents' lifestyles, leading to a decrease in basin water pollution and an alleviation of water quality deterioration. The response increased from 0.0527 to 0.1665, indicating relatively significant growth. These results suggest that measures taken by the government and society to address water resource issues have been effective, resulting in improvements in both the quantity and quality of water resources and an enhancement of water resource security levels. The evaluation value for impact fluctuated from 0.0261 to 0.0349, indicating a standardized industrial wastewater discharge volume and an improvement in water resource security conditions. The evaluation value for state initially decreased from 0.1633 to a minimum of 0.0656 before increasing to approximately 0.17. These results suggest that, considering indicators such as per capita sewage discharge and per capita water consumption, the status of water resources initially declined but gradually improved after governance measures were implemented.

In summary, from 2010 to 2019, the improvement in water resource security in the YRB can be attributed mainly to the enhancement of driving forces and response indicators. Economic and social development has provided ample assurance for water resource security, while water resources have imposed constraints on economic and social development to a certain extent. In the YRB, the current governance of water resources has reached a relatively high level, making it challenging to achieve significant breakthroughs in the future. The efficiency of water use in the existing industrial structure is difficult to substantially improve. Therefore, adjusting the industrial structure to enhance water resource security is a future research focus. These findings align with the conclusions of other domestic scholars. For instance, a study by Xiaotao and Fa-wen^64^ revealed that water consumption per unit of production energy and agricultural production in the YRB contributed the same proportion of GDP. They argued that future water conservation efforts should focus on adjusting industrial structures and developing water-saving technologies. Another study by Wang Hao revealed that the water resource utilization efficiency in the YRB was second only to that in the Beijing-Tianjin-Hebei region^65^. These authors suggested that the potential for mitigating the contradiction between water supply and demand through deep water conservation is limited.

According to the above methods and steps, further calculations were conducted to determine the water resource ecological security status of each province in the YRB from 2010 to 2019, as shown in Tables 6 and 7. Information gleaned from Tables 6 and 7 suggests that the overall improvement in the water resource ecological security status of each province in the YRB from 2010 to 2019 was significant. There was a discernible improvement from 2014 to 2015, with a clear boundary line. Before 2015, the water resources in most areas were relatively sensitive, and some regions even experienced deterioration. However, after 2015, almost all areas reached subsafe or safe states.

**Table 6:** Calculation results of the water resource security status of each province in the YRB from 2010 to 2014.

Province (region)	2010	2011	2012	2013	2014
ShangHai	Deteriorating	Vigilant	Sensitive	Deteriorating	Sensitive
JiangSu	Sensitive	Vigilant	Sensitive	Sensitive	Vigilant
AnHui	Sensitive	Deteriorating	Sensitive	Sensitive	Vigilant
JiangXi	Subsecure	Sensitive	Vigilant	Sensitive	Sensitive
HuBei	Sensitive	Deteriorating	Sensitive	Sensitive	Vigilant
HuNan	Sensitive	Deteriorating	Sensitive	Sensitive	Vigilant
ChongQing	Sensitive	Sensitive	Sensitive	Sensitive	Safe
SiChuan	Sensitive	Deteriorating	Vigilant	Vigilant	Vigilant
YunNan	Vigilant	Sensitive	Sensitive	Sensitive	Sensitive
QingHai	Vigilant	Vigilant	Vigilant	Sensitive	Vigilant

**Table 7:** Calculation results of the water resource security status of each province in the YRB from 2015 to 2019.

Province (region)	2015	2016	2017	2018	2019
ShangHai	Subsecure	Subsecure	Subsecure	Subsecure	Safe
JiangSu	Subsecure	Safe	Subsecure	Subsecure	Subsecure
AnHui	Subsecure	Safe	Safe	Safe	Subsecure
JiangXi	Vigilant	Subsecure	Subsecure	Subsecure	Safe
HuBei	Subsecure	Safe	Safe	Safe	Safe
HuNan	Subsecure	Safe	Safe	Safe	Safe
ChongQing	Sensitive	Safe	Safe	Subsecure	Subsecure
SiChuan	Vigilant	Subsecure	Safe	Safe	Safe
YunNan	Vigilant	Subsecure	Safe	Safe	Subsecure
QingHai	Sensitive	Vigilant	Subsecure	Safe	Safe

Calculation results of the water resource security status of each province in the YRB from 2010 to 2019.

### Trends in water resource ecological security in the Yangtze River Basin

**Figure 4:**
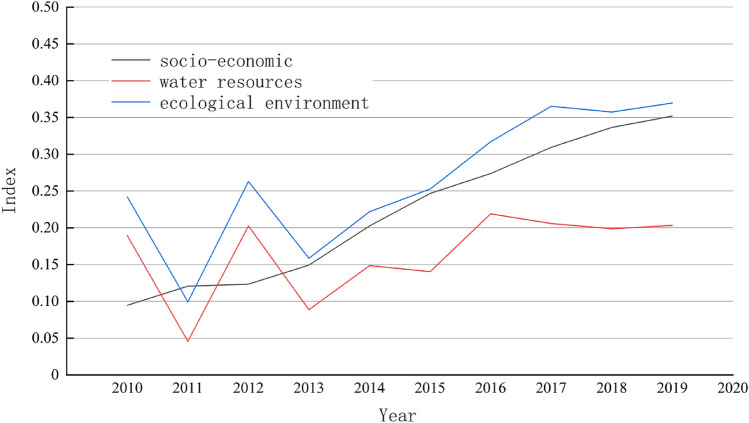
Development of the basic indices in the YRB.

According to Eq. (15), and by empirically examining the ecological status of water resources in the YRB from 2010 to 2019, the comprehensive levels of the ecological environment, socioeconomic development, and water resources in ten provinces of the YRB were obtained, as shown in Fig. 4.

The information gleaned from Table 4 suggests that the economic development in the YRB from 2010 to 2019 showed a positive trend, increasing from 0.09 to 0.35. This increase is attributed to the favourable current economic development environment and robust support from national directives. Policies such as the 2013 “Guiding Opinions on Building China’s New Economic Support Belt Based on the Yangtze River”, the 2018 speech at the Symposium on Deepening the Development of the YZR Economic Belt, the “Development Plan for the Huaihe River Ecological Economic Belt”, and the 2019 “Outline of the Development Plan for the Regional Integration of the Yangtze River Delta” have played crucial roles in driving industrial restructuring and achieving quality economic development in the YRB.

The ecological environment comprehensive level in the YRB exhibited a fluctuating development trend from 2010 to 2019, resembling an “M” shape, increasing from 0.24 to 0.37 with a relatively small amplitude. Ecological civilization construction, as a fundamental national policy, has provided important guidance for the economic development of the YRB. This development includes intensified efforts in the treatment of industrial pollutants and urban wastewater, along with increased levels of regional afforestation and greenery. Notably, significant improvements were observed in indicators such as per capita park green space, the urban green space ratio, and the harmless disposal of waste in the YRB in 2015.

The comprehensive level of water resources in the YRB increased slightly from 0.19 to 0.20 from 2010 to 2019. Although there was an upwards trend, the magnitude of the increase was minimal, indicating an unfavourable water resource status in the YRB. The primary factor in this slight increase is the accelerated consumption of water resources. As a part of the ecological environment, a decrease in the comprehensive level of water resources is also an important factor restricting the overall improvement of the ecological environment. In future development, the YRB should leverage favourable national policies to promote breakthrough development in the regional economy. Simultaneously, efforts should be intensified towards the protection and management of regional water resources and the ecological environment, striving to enhance the comprehensive level of water resources and the ecological environment.

**Figure 5:**
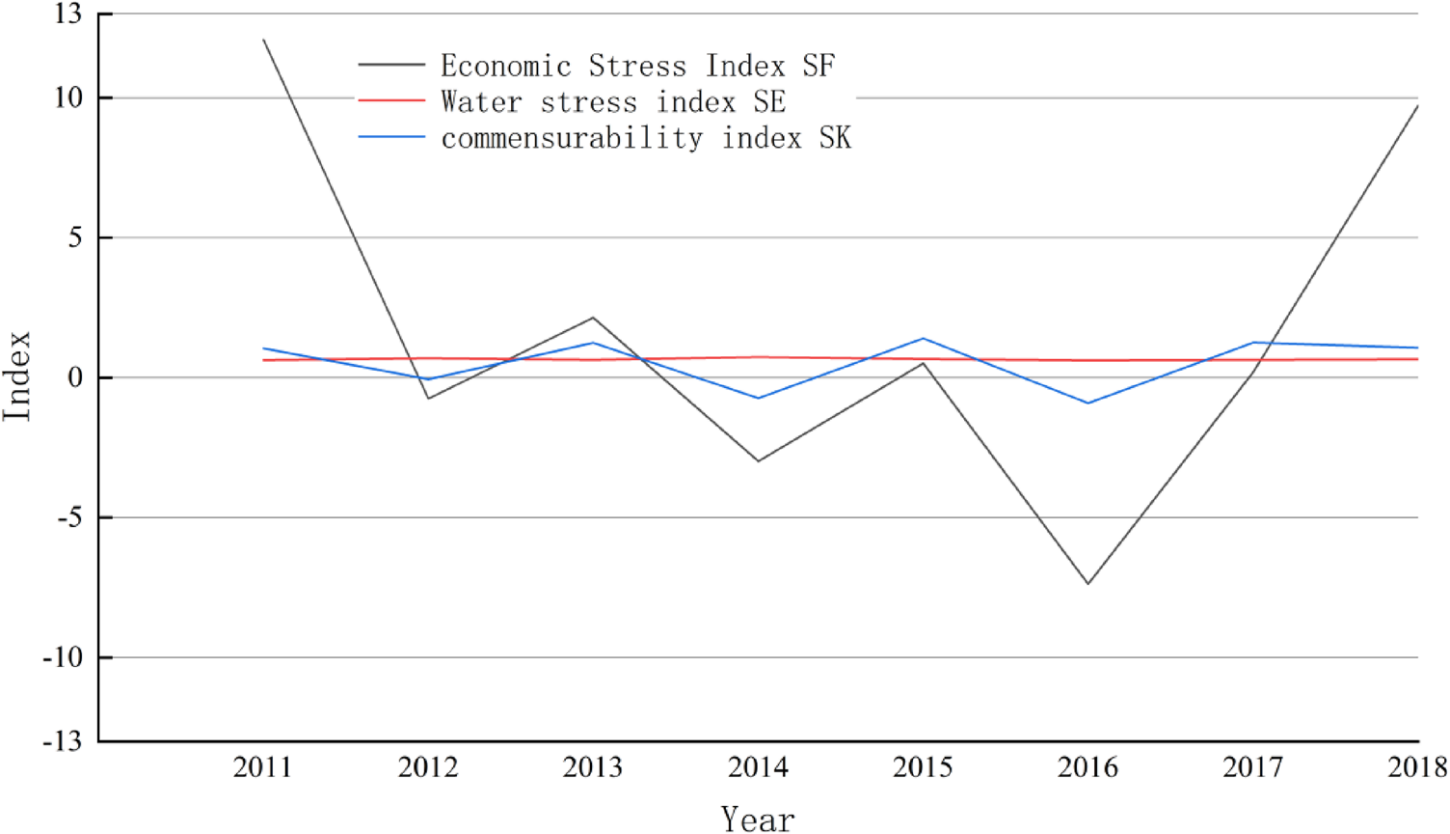
Development status of comprehensive indices in the YRB.

Based on the previously calculated comprehensive socioeconomic, ecological environment, and water resource levels, the stress indices for socioeconomic and water resources, as well as the symbiotic index for the YRB during the years 2010–2019, were computed, and the results are presented in Fig. 5.

Figure 5 clearly shows that, except for the years 2012, 2014, and 2016, the impact of water resources on the socioeconomy remained consistently positive, indicating that during this period, water resources positively contributed to economic growth. The water resources force index has been consistently positive in recent years, signifying the promotion by socioeconomic development, with a relatively minor hindrance from socioeconomic development during this period. The symbiotic index values between the two factors were 1.05, 1.24, 1.40, 1.26, and 1.07 in the years 2011, 2013, 2015, 2017, and 2018, respectively, reaching an optimal state of mutual benefit and symbiosis. However, a slight decline was observed in subsequent years, suggesting the need for further improvement.

## Discussion

### Spatial pattern analysis of water resource ecological security in the Yangtze River Basin

**Figure 6:**
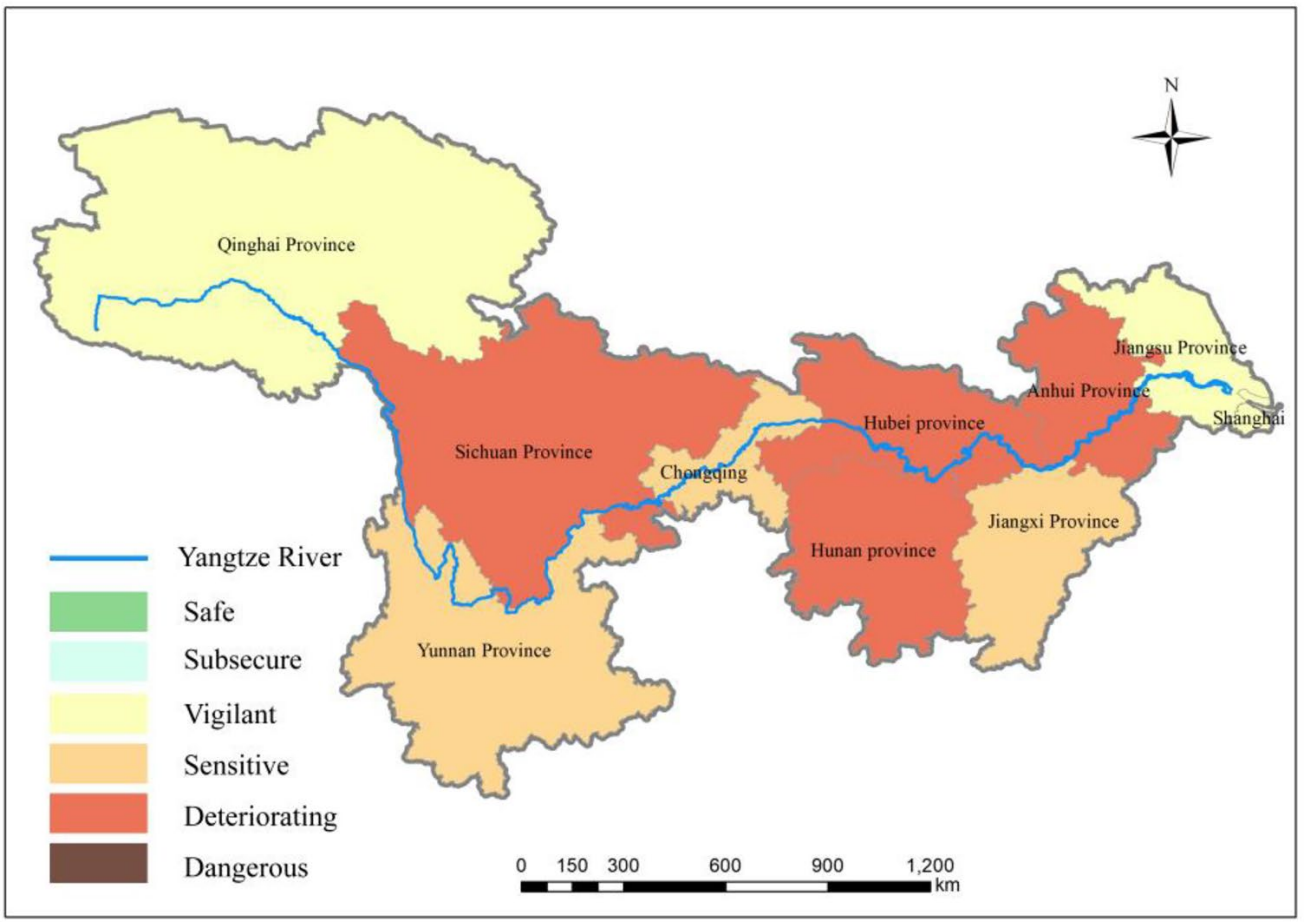
Ecological security status of water resources in the YRB in 2011(map were generated with software ArcMap10.4 http://www.esri.com/).

**Figure 7:**
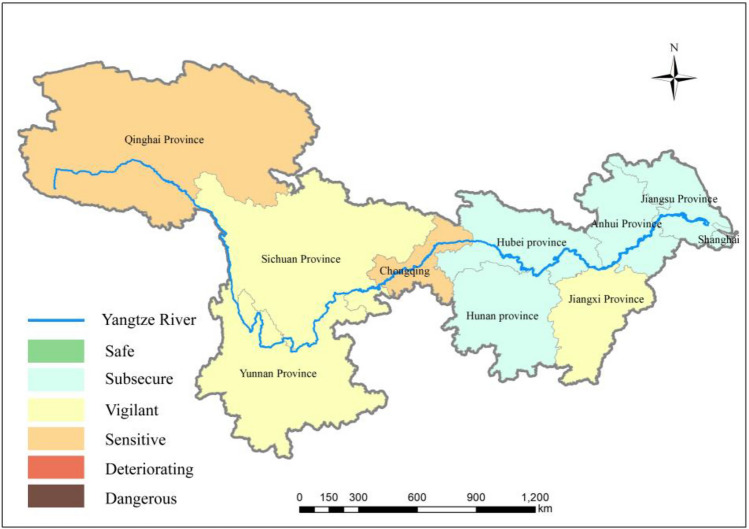
Ecological security status of water resources in the YRB in 2015(map were generated with software ArcMap10.4 http://www.esri.com/).

**Figure 8:**
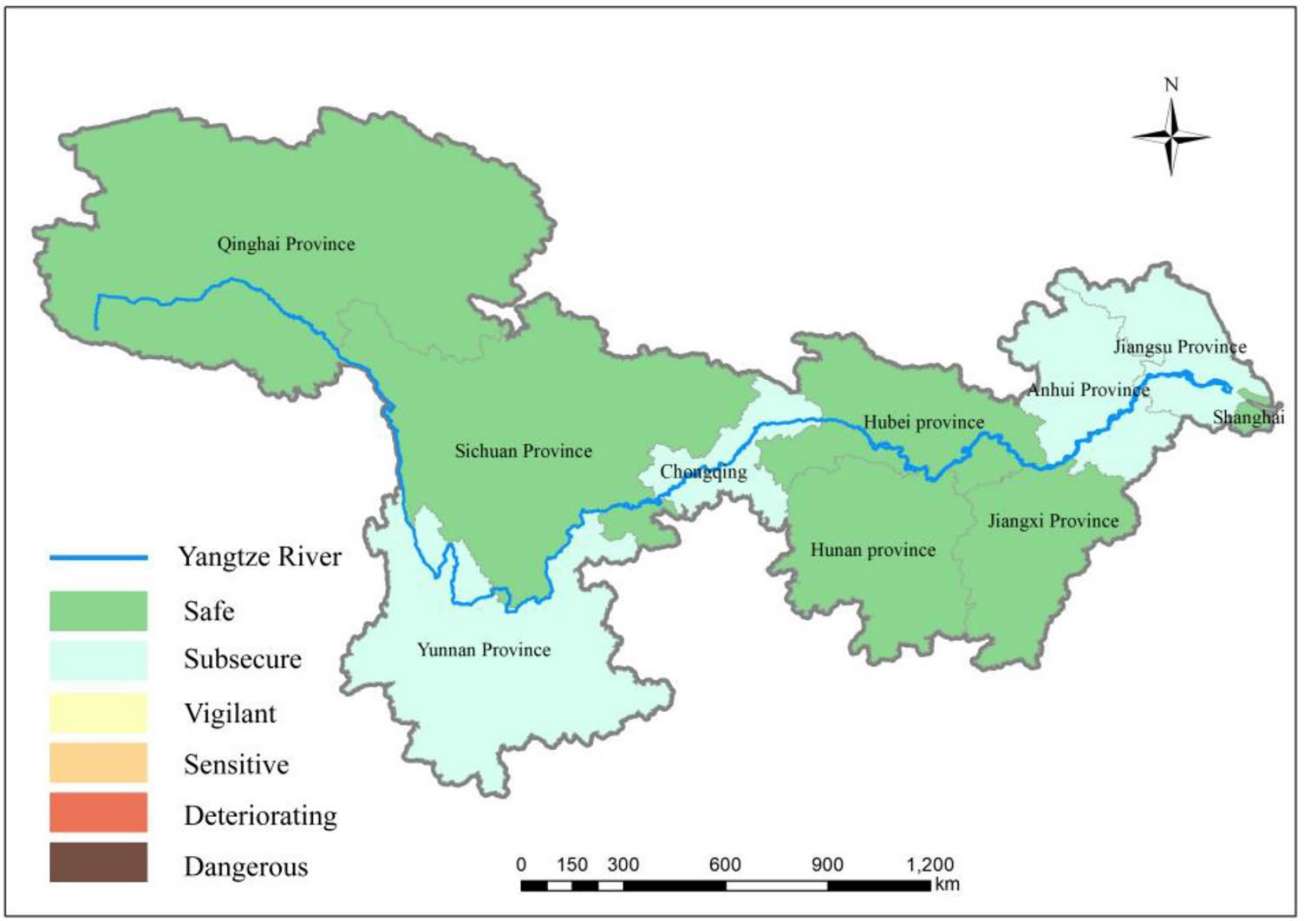
Ecological security status of water resources in the YRB in 2019(map were generated with software ArcMap10.4 http://www.esri.com/).

Using the ArcGIS10.4 tool, which is provided by the Environmental Systems Research Institute, Inc (commonly known as ESRI), several representative years were selected to visualize the ecological security status of water resources in the YRB. The computational results are visualized in Figs. 6, 7 and 8.

According to the division standards for administrative regions along the YZR in 2014, the YRB studied in this paper can be categorized into three main regions: the upper, middle, and lower reaches. The upper reach includes three provinces: Qinghai, Sichuan, and Yunnan. The middle reach comprises four provinces and municipalities: Chongqing, Hunan, Hubei, and Jiangxi. The lower reach consists of three provinces and municipalities: Anhui, Jiangsu, and Shanghai.

Figures 6, 7 and 8 show that from 2011 to 2019, the overall ecological security status of water resources in the YRB transitioned from “deteriorating,” “sensitive,” and “vigilant” states to “subsecure” and “safe” states. The range of comprehensive evaluation values for water resource ecological security (hereafter referred to as evaluation values) increased from 0.16–0.64 to 0.64–1.

As illustrated in Fig. 6, notable disparities were present in the distribution of the ecological security status of water resources among provinces and municipalities in the YRB, with the ecological security status of water resources in the upper and lower reaches of the YZR notably superior to that in the middle reaches. The data indicate that the water resource utilization efficiency levels in the upper and lower reaches of the YZR were greater than that in the middle reaches in 2011, exhibiting a pattern of high efficiency at both ends and lower efficiency in the middle. Regions with high comprehensive water resource utilization efficiency are mainly concentrated in the upper and lower reaches of the YZR.

Although the upstream regions have limited economic strength, they also have relatively fewer water-intensive industries. Meanwhile, these regions actively respond to green development policies and prioritize energy conservation and environmental protection industries. Underdeveloped regions can also achieve higher water resource efficiency by controlling total water consumption and improving the output of water per unit used.

The areas with low comprehensive utilization efficiency of water resources are primarily concentrated in the middle reaches of the YZR, where the proportions of traditional industries such as steel, chemicals, and nonferrous metals are relatively large, leading to high industrial water consumption and consequently the lowest efficiency in water resource utilization. Provinces such as Hunan and Hubei, with large populations and rapid economic development, exhibit high demands for water resources, resulting in increased regional water resource consumption and persistently high per capita sewage discharge indicators.

The downstream regions of the YZR boast strong economic progress, with high levels of industrial technological innovation and governance capabilities. This region exhibits the highest level of economic development, which can drive improvements in the utilization efficiency of water resources. Consequently, Shanghai and Jiangsu provinces have the highest water resource utilization efficiency. As a result, the ecological security status of water resources in Shanghai has improved rapidly.

As shown in Fig. 7, in 2015, the overall ecological security status of water resources notably improved in the YRB. The fundamental reason for this improvement is that in recent years, regions across the basin have recognized the importance of the ecological environment for overall development. They have gradually undertaken regional industrial restructuring and upgrading and accelerated urbanization and simultaneously emphasized the preservation of water resources and the environment. The three major regions exhibit regional disparities in water resource utilization efficiency due to differences in geographical environment, economic foundation, and industrial structure. In terms of the total water consumption of each province and municipality, agricultural water usage accounts for more than half of the total water consumption, which is significantly greater than the water usage in the industrial, domestic, and ecological sectors. However, compared to other industries' output values, the overall water resource utilization efficiency in agriculture is lower. Therefore, regions with greater proportions of primary industry output tend to have lower water resource utilization efficiency.

The industrialization level in the upstream regions is relatively low, with relatively outdated production technologies. As industrialization progresses, the negative impact on water resources' ecological security is gradually increasing. The industrialization in the middle and lower reaches of the YZR has reached relatively high levels. Control measures have been gradually implemented to manage the resource consumption and environmental pollution generated during the industrial development process. With advancements in technology, the negative impact on water resource ecological security is gradually diminishing. Among these provinces, Hunan Province and Hubei Province in the middle reaches of the YZR experienced the greatest increases in water resource ecological security status, transitioning from “deteriorating” to “subsecure.” The regions in the middle reaches emphasize considering the resource and environmental carrying capacity to ensure the coordination between water resource allocation and regional sustainable development, achieving rational distribution and efficient utilization of water resources within the region.

The lower reaches of the YZR are characterized by developed economies, advanced technologies, and high levels of both urbanization efficiency and water resource efficiency, maintaining harmonious development. This region exhibits the strongest economic development and hosts the highly integrated YZR Delta urban agglomeration. With a solid foundation in secondary and tertiary industries, high levels of technological innovation, and openness, the overall ecological security status of water resources in this region is at a relatively high level.

Across the provinces and municipalities in the YRB, efforts have been intensified to control the discharge of pollutants such as phosphorus, leading to reduced pollutant emissions and improved water quality. Moreover, improvements in water resource allocation have been made, reducing the risks associated with pollution factors through increased water volume and dilution effects, thereby ensuring the supply and safety of drinking water downstream of Shanghai. The stable proportion of GDP in the YZR Economic Belt indicates a balanced relationship between economic development and the ecological protection of water resources. While maintaining economic growth, downstream cities also prioritize environmental protection and water resource management.

Figure 8 clearly shows that the overall ecological security status of water resources in the YRB has been developing at an accelerated pace, trending towards overall coordinated development by 2019, with mutual promotion between socioeconomic and water resources. This trend can be attributed to various factors. This positive influence is exemplified in agricultural water use efficiency, which has improved in recent years due to various factors, such as changes in agricultural production methods, organizational structures, cropping patterns, and water-saving practices. As a result, the negative impact of the proportion of the output value of the primary industry on water resource efficiency has been mitigated.

However, despite efforts, China still faces serious water pollution issues, with poor water environmental quality and significant pollution discharge loads from industrial, agricultural, and domestic sources. These factors pose severe challenges to the ecological security of water resources. To address these challenges, China has formulated a series of plans aimed at strengthening water pollution prevention and control and ensuring national water resource ecological security. These plans were officially announced and implemented after 2015.

Based on the analysis results, each province and city in the YRB should embrace a people-centred approach to new urbanization and the scientific development concept of water resource protection and utilization. While focusing on promoting new urbanization construction, efforts should be intensified to enhance ecological environmental protection and explore new paths for coordinated regional economic development and resource utilization. Provinces and cities should rely on the golden waterway of the YZR to establish cross-regional and cross-provincial basin cooperation mechanisms and long-term mechanisms, actively promoting coordinated development among the three major regions of the YRB.

Against the backdrop of the global environmental crisis, the Lancang-Mekong River, as Asia’s largest transboundary river, also faces certain water security issues. Specifically, the “status” of water resources is relatively low, as manifested by the polluted state of the water quality of the river. Additionally, factors such as the uneven distribution of precipitation within the year and the weakness of storage facilities such as wetlands and reservoirs contribute to seasonal water shortages and serious water disasters in the basin. Moreover, the response levels of basin countries are limited, and there is room for improvement in the level of water resource management. Countries in the Lancang-Mekong River Basin are in a stage of rapid economic and social development, and population growth, economic activities, and changes in land use (such as urbanization) will have direct or indirect impacts on water resources in the basin. The Ganges River Basin faces similar ecological and environmental problems. In recent years, India’s economic prosperity and urbanization process have had significant impacts on the Ganges River Basin. Soil erosion and insufficient drinking water under population pressure have plagued the people of the Ganges River Basin. Additionally, the serious problem of surface water pollution caused by the discharge of industrial and domestic wastewater has led to a certain degree of land salinization.

Climate change, land use, human consumption of water resources, and government management of water resources are all factors that can directly or indirectly affect the water security situation in a region. Given that the Lancang-Mekong River spans China and five Southeast Asian countries, its water resource ecological security is particularly influenced by socioeconomic factors. Therefore, we believe that the methods we propose are equally applicable to the evaluation of water resource ecological security in this basin. By introducing the Lotka–Volterra symbiotic model and using the DPSIR model to construct a system of evaluation indicators for the symbiosis between socioeconomic factors and water resources in the study area, this system will help us to thoroughly assess the water resource ecological security of the Lancang-Mekong River Basin and provide a scientific basis for the implementation of region-specific water security strategies. These approaches are highly important for promoting regional sustainable development and maintaining basin ecological security.

## Conclusion

Research has revealed that over a decade ago, the water resource ecological security status in the YRB initially fell within a relatively poor range. However, with close attention from the government and the implementation of various regulations, as well as active participation from the public in protecting the YZR, the water resource ecological security status in the YRB has improved rapidly. It is now generally maintained at levels of safety or near safety, with prospects for further improvement in the future. Comprehensive analysis of data from 2010 to 2019 revealed continuous trends in improvement in water resource security. To further enhance water resource security, we propose the following recommendations:

1. **The industrial structure should be adjusted to achieve sustainable utilization of water resources.** Governments should strongly support the green economy and environmental protection industries by providing tax incentives for enterprises, encouraging them to invest in water resource management and protection projects. By establishing corresponding financial funds and reward mechanisms, more social forces can be guided to participate, achieving a mutually beneficial outcome for water resource security and economic development. The Chinese government has called for all citizens to actively respond to carbon peak and carbon neutrality strategies and has formulated specific and feasible emission reduction plans. Enterprises are encouraged to adopt clean production technologies to improve resource utilization efficiency and achieve carbon emission reduction goals. There should be a focus on strengthening sewage resource utilization, integrating atypical water sources into unified water resource allocation, and encouraging locations with the necessary conditions to fully utilize unconventional water sources. Water-deficient cities should actively expand the scale and scope of recycled water utilization. The principles of demand-driven supply, water quality division, and local utilization should be followed to promote the use of recycled water in industrial production, municipal miscellaneous use, land greening, ecological replenishment, and other areas.
2. **Focusing on agricultural water use and preventing water source pollution.** As one of the main rice-producing regions in China, to further enhance water resource security in the YRB, agricultural measures should be taken. With respect to water conservation, water-saving irrigation techniques combined with smart irrigation systems should be adopted to achieve precise irrigation and improve water resource utilization efficiency. Moreover, enhancing rainwater collection and utilization by establishing rainwater collection systems and storing water for agricultural irrigation can effectively utilize rainwater resources and alleviate irrigation pressure during the dry season.

Agricultural pesticide use is also an issue that cannot be ignored. Excessive use and improper handling of pesticides can often lead to serious water pollution, posing a threat to the water resource security of the YRB. To address this issue, we need to strengthen pesticide use management, promote scientific pesticide application techniques, reduce excessive pesticide use, raise farmers' environmental awareness to prevent pesticide waste from being directly discharged into water bodies, and strengthen water quality monitoring and treatment to promptly detect and address pesticide pollution problems.

3. **Improve people’s education level and strengthen environmental awareness.** As people's living standards and education levels improve, concerns about ecological water security have increased, and higher demands are being placed on water safety and quality. The incomplete assessment and mismanagement of water resources, coupled with wasteful practices, have led to water resources becoming uncontrollable variables. Recognizing, measuring, and expressing the value of water and incorporating it into decision-making processes are particularly important against the backdrop of increasingly scarce water resources, population growth, and the pressures of climate change. It is essential to achieve sustainable and equitable water resource management and meet the development goals of the United Nations' 2030 Agenda.
4. **Actively participate in international ecological construction.** According to Maximo Torero of the FAO, strengthening water resource protection and management requires enhanced cooperation among countries, the integration of various stakeholders' interests, multipronged approaches, and the consideration of social, economic, and environmental factors. It also involves a focus on technology, legal frameworks, and overall policy environments. We recommend that governments actively engage in international cooperation projects, sharing experiences and technologies in managing water resources in the YRB while drawing lessons from successful ecological initiatives in other countries. Such cross-border collaboration can foster global ecological sustainability, address global environmental issues collectively, share innovative technologies and research achievements, and achieve global governance of ecological environments.

## Data Availability

Our data is sourced from the provincial data in the China Statistical Yearbooks from 2011 to 2019 published by the National Bureau of Statistics of China (https://www.stats.gov.cn/sj/ndsj/), as well as the Water Resources Bulletins (http://www.mwr.gov.cn/sj/tjgb/szygb/). Figures 6, 7, and 8 were created by us using ArcGIS 10.4 software, which is provided by the Environmental Systems Research Institute, Inc. (commonly known as ESRI). Our vector boundary data and the Yangtze River data are sourced from the National Catalogue Service For Geographic Information (www.webmap.cn), using the 1:1,000,000 public version of basic geographic information data (2021). The tiled data is processed according to GB/T 13989-2012 “National Fundamental Scale Topographic Map Tiling and Numbering”.
